# Informing patients of familial diabetes mellitus risk: How do they respond? A cross-sectional survey

**DOI:** 10.1186/1472-6963-8-37

**Published:** 2008-02-07

**Authors:** Nadeem Qureshi, Joe Kai

**Affiliations:** 1 Division of Primary Care, School of Graduate Entry Medicine and Health, University of Nottingham, Derby City General Hospital, Uttoxeter Road, Derby, DE22 3DT, UK

## Abstract

**Background:**

A strong family history of type 2 diabetes mellitus (DM) confers increased DM risk. This survey analysis determined whether patients who were informed by their doctors of familial DM risk acknowledged that risk and took steps to reduce it.

**Methods:**

We conducted an analysis of the National *Health Styles 2004 *mail survey. All non-diabetic participants who responded to the question of whether their doctor had or had not informed them of their familial DM risk (*n *= 3,323) were compared for their risk-reducing behaviour and attitude to DM risk.

**Results:**

Forty-one percent (*n *= 616) of the question responders that had DM family histories were informed by their doctors of their familial risk; the chance of being informed increased with the number of relatives that had the disease. Members of the informed group were more likely than those in the non-informed group to report lifestyle changes to prevent DM (odds ratio [OR] 4.3, 95% confidence interval [CI] 3.5–5.2) and being tested for DM (OR 2.9, 95% CI 2.4–3.6), although no significant improvement occurred in their U.S.-recommended exercise activity (OR 0.9, 95% CI 0.7–1.1). Overall, informed responders recognised both their familial and personal DM risk; most discussed diabetes with their family (69%), though less so with friends (42%); however, 44% of them still did not consider themselves to be at risk.

**Conclusion:**

Responders who were informed by their doctors of being at familial DM risk reported greater incidences of lifestyle changes, DM screening, and awareness of risk than non-informed responders. Doctors were more likely to inform patients with stronger DM family histories. Identifying this higher risk group, either in isolation or in combination with other recognised risk factors, offers doctors the opportunity to target limited health promotion resources efficiently for primary DM prevention.

## Background

Diabetes mellitus (DM) rates continue to rise and are predicted to reach epidemic proportions among the U.S. population within the next 50 years [1]. This dramatic rise is a global phenomenon. Recognized, modifiable risk factors for type 2 DM include a diet high in fat and sugar, obesity, and lack of exercise. Non-modifiable risk factors include age, ethnicity, and family history. A growing body of evidence indicates that DM can be prevented or its onset delayed through lifestyle modification [2]. In the U.S., the Diabetes Prevention Program recruited patients into an exercise and weight loss program [3] that resulted in a 58% reduction in the incidence rate of diabetes [4]. Similar results were achieved in a randomized controlled trial of overweight subjects with impaired glucose tolerance in Finland [5].

One approach that seems to improve the effectiveness of lifestyle interventions is to involve family doctors in providing advice and reinforcing health promotion activities [6]. As part of their assessment of DM risk factors for patients, doctors can use family history of DM to identify patients at increased risk of developing diabetes [7]. A parental family history increases a person's risk of developing DM by two to five times [8]. The purpose of this study was to determine whether being informed by a doctor of increased DM risk because of family history is associated with improvement in risk-reducing behaviors. We also sought to assess whether those informed of their familial risk or not may differ in their attitudes about diabetes prevention and risk awareness.

## Methods

Relevant programs of the Centers for Disease Control and Prevention (CDC) developed questions related to family history and diabetes for inclusion in the national cross-sectional *HealthStyles 2004 *mail survey, a subset of the two-part *Styles 2004 *consumer survey that was administered by Synovate, Inc. *HealthStyles 2004 *collected data on health-related attitudes and behaviors among the U.S. adult population aged 18 and older. To ensure that the *HealthStyles *survey is representative of the whole population, the survey oversamples low income and minority groups and results are weighted to US census benchmarks. The *HealthStyles 2004 *mail survey consists of 356 questions in 12 sections. During 2004, the survey was sent to a random sample of 6,175 households, of which 4,345 agreed to participate, resulting in a response rate of 70%.

The survey collected information on selected socio-demographic variables, such as age, gender, income, educational status, and self-reported racial group. Data were also collected on diabetes and cardiovascular disease risk factors, such as obesity, and smoking history, which were then included in the analyses. Questions related to familial DM history are listed in Additional file 1. These include: doctor advising respondent of familial risk, presence of diabetes in parents and siblings, as well as total number of affected paternal and maternal relatives (i.e., aunts, uncles, and grandparents). Additional questions were asked if respondents have actively collected family history; beliefs about familial DM risk; lifestyle changes, such as diet and exercise, to reduce risk; whether the respondent had been tested for and was aware of his or her blood sugar results; and whether the respondent had discussed diabetes with family, friends, and religious advisors. Current reported exercise level was analyzed against U.S. recommendations (i.e., 3 days per week of vigorous exercise for 20 minutes *or *5 days per week of moderate exercise for 30 minutes) [9]. In addition, perceived DM risk was explored through several questions that noted the respondents' perceptions of their personal and familial DM risk as well as their attitudes about preventing risk.

Univariate analysis was conducted to obtain the crude estimate of the main independent variable of interest for each response variable. Unconditional maximum likelihood estimation of the multivariate logistic regression was also performed for each response variable by using backward elimination method at a 0.05 significance level. The adjusted odds ratios (ORs) and 95% confidence intervals (CIs) were computed for all significant variables, adjusting for basic demographic variables and selected health indicator variables in the model specifically, age, sex, racial group, income level, marital status, education status, self-reported obesity, and number of primary doctor visits.

## Results

Of the 4,345 respondents, 3,683 (85%) reported that they did not have diabetes. Of these, 3,323 (90%) respondents answered the question of whether their doctor had or had not informed them if they were at greater risk for diabetes because of a family history of DM. The remaining analyses presented in this paper are based on these 3,323 respondents. (No significant difference in the selected socio-demographic variables was found between the 90% of responders who completed the question on being advised by their doctor of their familial DM risk and the other 10% who did not respond to the question, except that the responders were more likely than the small proportion of non-responders to have a college level education or higher (χ^2 ^= 13.1, *P *= 0.0003). Table 1 compares the distribution of selected socio-demographic variables of the respondents who said their doctor informed them that they were at greater risk because of their family history (referred to as the *informed group *in subsequent text) to the respondents who were not informed of increased risk due to family history.

**Table 1:** Characteristics of question respondents

	Informed of familial DM risk by doctor	
	**YES **(*n *= 709) *n *(%)	**NO **(*n *= 2,614) *n *(%)
Age (years)		
18–34	153 (22)	498 (19)
35–54	412 (58)	1,369 (52)
55+	144 (20)	747 (29)
		
Gender		
Male	256 (36)	1,177 (45)
Female	453 (64)	1,437 (55)
		
Educational status*		
- Up to high school	224 (32)	731 (28)
- ≥ College	461 (65)	1,754 (67)
		
Income status		
< $25 k	203 (29)	656 (25)
$25 k–$59 k	245 (34)	916 (35)
$60 k +	261 (37)	1,042 (40)
		
Marital status*		
Ever married	621 (88)	2,273 (87)
Never married	80 (11)	310 (12)

DM, diabetes mellitus.

*The categories do not add up to Total Question Responders due to missing values.

Of the 3,323 responders, 21% stated that their doctors informed them of an increased DM risk because of their family history. Adults older than age 55 had a lower prevalence of being informed than younger respondents (20% of informed group aged 55 years and older, compared to 29% of uninformed group). Proportionately more women reported being informed (64% of informed vs. 55% of uninformed group). The prevalence of being informed varied little by education level, income, or marital status.

Table 2 demonstrates the profile of responders in relation to DM risk factors. The informed group was more likely than the uninformed group to report being obese (21% vs. 12%, respectively), being of a race other than white, and having a family history of DM (87% vs. 34%, respectively). Reviewing in more detail the self-reported family history of diabetes in the survey, 59% (*n *= 887) of responders with any family history of DM had not been informed of DM risk by their doctor. Being informed of familial risk was associated with a greater number of relatives being affected (Table 3). When only specific relatives were identified, the proportion of responders informed of risk was higher if both first- and second-degree relatives were affected (67% [*n *= 335]), but was lower if only first-degree relatives (38% [*n *= 101]) or only second-degree relatives (31% [*n *= 200]) had DM. In contrast, 3% (40) of respondents with no family history reported being informed by their doctors as being at familial DM risk.

**Table 2:** Risk factors for diabetes mellitus

	Informed of familial DM risk by doctor	
	**YES **(Total *n *= 709) *n *(%)	**NO **(Total *n *= 2,614) *n *(%)
**Modifiable**		
		
Self-reported obesity		
Yes	151 (21)	324 (12)
No	558 (79)	2,290 (88)
		
**Non-modifiable**		
		
Racial groups		
White	468 (66)	1,885 (72)
Black	102 (14)	287 (11)
Hispanic	103 (15)	267 (10)
Other	36 (5)	175 (7)
		
Family history*		
Yes	616 (87)	887 (34)
No	40 (6)	1,209 (46)

DM, diabetes mellitus.

*The Family History category does not add up to Total Question Responders due to missing values.

**Table 3:** Self-reported diabetic family history of respondents informed of familial DM risk

Variable	Odds Ratio (OR) of being informed of familial DM risk by doctor	
	Adjusted OR	95 CI%
***Level of Family History Recording***		
		
**Total relatives with DM (*n*)**		
		
0	1	referent
1	7.0	5.3, 9.4
2	12.1	8.8, 16.7
3 +	39.0	28.8, 52.8
		
**Relatives with DM**		
		
None	1	referent
Second degree only	8.7	6.0, 12.6
First degree only	17.5	11.2, 27.3
		
Both first and second degree	51.6	35.5, 74.9

Adjusted for age, sex, racial group, income level, marital status, education status, self-reported obesity, and number of primary care doctor visits.

**Figure 1:**
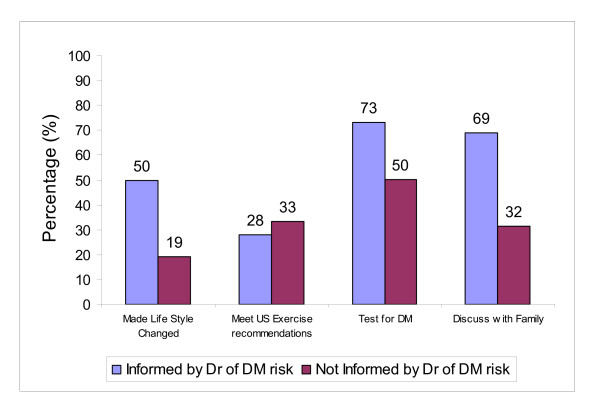
Behavior of question respondents: comparing those informed of familial diabetes mellitus (DM) risk against those not informed.

Figure 1 demonstrates the proportion of informed and uninformed respondents that reported making behavior changes to prevent DM. Up to 50% of the informed group reported lifestyle changes to prevent DM, compared to 19% of the uninformed group (OR 4.3, 95% CI 3.5–5.2). Although Figure 1 also demonstrates that 33% of the uninformed group met U.S. exercise recommendations, compared to 28% of the informed group, this difference was not statistically significant when controlling for other variables, as demonstrated by the adjusted OR in Table 4.

**Table 4:** Characteristics of respondents informed of familial DM risk

Variable	Odds Ratio (OR) of being informed of familial DM risk by doctor	
	Adjusted OR	95 CI%
		
***BEHAVIOUR***		
		
**Lifestyle changes to prevent DM**		
No	1	referent
Yes	4.3	3.5, 5.2
		
**Meet U.S. exercise recommendations**		
No	1	referent
Yes	0.9	0.7, 1.1
		
**Tested for DM in past 12 months**		
No	1	referent
Yes	2.9	2.4, 3.6
		
**Actively collect family history**		
No	1	referent
Yes	2.7	2.2, 3.2
		
**Discuss diabetes with family**		
No	1	referent
Yes	4.7	3.9, 5.6
		
**Discuss diabetes with friend**		
No	1	referent
Yes	3.1	2.5, 3.7
		
***ATTITUDE***		
		
**Believe at DM risk**		
Disgree	1	referent
Agree	7.4	6.1, 8.9
		
**Believe family is at risk of type 2 DM**		
Disgree	1	referent
Agree	5.6	4.7, 6.8
		
**Can delay or prevent DM with weight loss/physical activity**		
Disgree	1	referent
Agree	1.9	1.6, 2.3
		
**Aware of blood sugar level**		
No	1	referent
Yes	2.6	2.1, 3.2

Adjusted for age, sex, racial group, income level, marital status, education status, self-reported obesity, and number of primary care doctor visits.

Further, the informed group was more likely than the uninformed group to recognize their personal DM risk (56% vs. 14%, respectively). However, 44% of the informed group still considered themselves not to be at risk. The magnitude of the difference between the informed and uninformed groups was less for attitudes to delay or prevent DM through lifestyle changes (72% vs. 58%, respectively) and awareness of blood sugar levels (30% vs. 16%, respectively). The multivariate analysis, presented in Table 4, also demonstrated the significant association between risk-reducing behaviour and being informed of familial diabetic risk, except for meeting U.S. exercise recommendations (as discussed above).

### Discussion of risk with family or friends

Sixty-nine percent (489) of all informed respondents, compared with 32% (824) of uninformed respondents, discussed diabetes with their family. Among informed respondents, the majority (78%) of those of Hispanic (*n *= 81) and black (*n *= 80) origin reported that they had discussed diabetes with their family, compared to 66% (*n *= 309) of whites. This finding did not reach statistical significance when adjusted for other factors (compared to white group: Hispanic adjusted OR 1.5, 95% CI 0.9–2.6; black adjusted OR 1.5, 95% CI 0.9–2.5). A less dramatic difference was found between the informed (42% [*n *= 295]) and uninformed (30% [*n *= 793]) respondents who discussed diabetes with friends. Among informed respondents, 50% (51) of the Hispanic group discussed diabetes with a friend, compared to 47% (48) of the black and 39% (184) of the white groups. In logistic regression analysis, racial group had no statistically significant effect in both univariate and multivariate analysis (compared to white group: Hispanic adjusted OR 1.5, 95% CI 1.0–2.3; black adjusted OR 1.4, 95% CI 0.9–2.1). No statistical significant differences were found between reported racial identity of informed respondents and actively collecting family history information: compared to the group of informed whites, the adjusted OR for informed blacks was 1.2 (95% CI 0.9–1.5) and for informed Hispanics was 1.0 (95% CI 0.8–1.2).

## Discussion

To our knowledge, this is the first cross-sectional survey of the general population to explicitly enquire about the relationship between physicians' advice about familial risk and lifestyle changes and actual DM screening. This aspect of the diabetic family history's clinical utility has not previously been explored. Overall, the respondents who were informed of their familial DM risk by their doctors reported more general lifestyle changes to reduce their risk of diabetes. However, no significant difference was found in exercise activity between informed and uninformed groups.

Previous findings indicate that patients at perceived DM risk are more engaged in controlling their weight [10] and are more likely to prevent DM by changing their diet than by exercising [11]. Up to half of respondents in this survey who were identified and informed by their doctors as being at DM risk indicated change in their lifestyle behavior, suggesting there may be considerable potential to enhance such change in this high-risk group. Health promotion resources should be targeted to support these individuals. It is nevertheless heartening that, consistent with other studies, people at familial risk for diabetes were more likely to engage in DM testing [12].

Given these findings, the potentially advantageous consequences of informing patients of familial DM risk may be considerable. Even a small change in behavior at a community or individual level may have a dramatic impact at a population level. For example, in considering a different health promotion message, doctors advising patients to stop smoking led to around a 2.5% cessation rate. The relevant Cochrane systematic review states that this modest level of change would have a significant public health impact for a such a low-cost intervention [6]. For DM, at an individual level, Wing at al. have noted that at-risk overweight patients who reduce their body weight by 4.5% can experience up to a 30% reduction in their risk of developing diabetes over a 2-year period [13].

Arguably more promising than lifestyle changes suggested in this survey is the finding that informed group members appeared more aware than uninformed group members of their DM risk and of diabetes screening. Informed group members were also more likely to collect family history information. This high level of awareness could be translated into proactive health promotion messages at appropriate "teaching moments" in the patients' interactions with their primary care providers. Previous research suggests that a patient's perception of risk alone may not be sufficient to lead to lifestyle changes and self-surveillance [10], but the addition of the doctor's comments can provide the extra impetus for change. On the other hand, despite being informed of familial DM risk, nearly half of the informed respondents still did not consider themselves at risk.

Almost 60% of responders who reported DM family histories on the survey were not informed of their DM risk by their doctors. This finding may simply mean that the respondents did not report their family histories to their doctors or that the doctors either judged the information as indicating minimal familial risk or where unaware of the implications of this family history. Our data suggests the chance of being informed increased with the increase in the number and type of relatives affected. However, this finding may still represent a significant proportion of at-risk individuals not informed of the relevance of their family history information and, thus, not offered DM screening. There are three further implications of these findings. Firstly, a patient's awareness of their family history, in its own right, may lead to risk-reducing behaviour (irrespective if they are informed by their doctor or not). By combining two subgroups in the "not informed" group (respondents with no family history and patients with a family history but not informed of their risk by doctor) the actual impact of being informed by doctor may be reduced. On the other hand, in the subgroup that have relayed their family history to their doctors but not informed by their doctor of familial risk, may consider their level of personal risk does not necessitate the need to improve their lifestyle. Further, the process of diabetes family history enquiry by the doctor may lead to patients adopting risk-reducing behaviour. Hence, for example, in the situation where respondents state in the survey that they do not have a family history, and the doctor had not enquired about a relevant family history, then they would not be exposed to this possible intervention. The level of detail to identify these implications could not be extracted from this survey.

The study is strengthened by *Health Styles 2004 *sampling which recruited respondents with a demographic profile and diabetes prevalence similar to the Behavioral Risk Factor Surveillance System (BRFS) and the general population [14]. However, as with most large population surveys, data are based on self-report. General statements about lifestyle changes may not provide an accurate assessment of either intent to change lifestyle or actual behavior changes. Moreover it was not possible to assess the temporal sequence of lifestyle modification and being informed of familial diabetic risk. Future surveys should also consider more focused enquiry on the exact nature of lifestyle change. A further aspect of self reported surveys is that respondents may attempt to keep responses consistent between questions, for example between risk awareness and lifestyle responses. This could be relevant when questions are grouped together (see Diabetes family history section of the *Health Styles 2004 *survey in Additional file 1), but in some instances the questions were in different sections making consistent desirable responses less likely, for example there were 48 questions separating the question about doctors informing responders of their familial risk and the exercise question. Despite these limitations, the findings are useful in generating hypotheses that can be explored in pragmatic intervention studies, in particular that being informed of familial diabetes risk can provoke lifestyle change to reduce risk of diabetes.

In addition to family history, ethnic or racial origin is a significant risk factor for DM. Among people born in the U.S. in 2000, Hispanics and blacks have a higher estimated lifetime risk of developing DM than non-Hispanic whites. There is a 45% estimated lifetime risk for Hispanic males, 53% for Hispanic females, 40% for black males, and 49% for black females compared to 27% for males and 31% for females in the non-Hispanic white population [15]. Previously, ethnicity and obesity have triggered diabetes screening by physicians. This is supported in this survey by proportionally more black, Hispanic, and self-reported obese respondents being informed by doctors of their familial risk. This result is consistent with Harwell et al.'s finding that respondents with a greater number of DM risk factors (including family history) are more likely to be advised by a health care professional about their risk [16]. However, in the future, a strong family history, in its own right, may trigger proactive screening for DM and, thus, delay or prevent the onset of DM in high-risk populations [17]. This low-cost approach to primary diabetic screening could be used in underserved and underinsured populations, such as disadvantaged and ethnic minority populations, that may not or cannot avail themselves of preventive services despite the fact that they are at greatest risk of multifactorial conditions like type 2 DM [18]. The potentially greater involvement of family and friends in patients' discussion of disease risk among minority populations identified in this study may hold promise. Here, the implementation of community health care workers, recommended by the Institute of Medicine, in conjunction with patients' support networks of family and friends, might facilitate behavioral changes in response to risk identification [19].

Examining family DM history may be a valuable approach for identifying patients at-risk for diabetes. In addition, this survey provides some indication that knowledge of family history may lead to patients adopting preventive measures. This forms a hypothesis for further testing. Alongside such future research, the clinical use of family history for diabetes can be further explored. Will doctors intervene if familial DM risk is targeted and identified? Current studies suggest rather limited intervention by doctors who opportunistically identify diabetic risk in their patients [12,20]. Further, if doctors offer patients lifestyle advice and glucose screening, will this lead to sustained improvement in modifiable risk factors and ultimately prevent DM [2]?

## Conclusion

Individual, in this cross-sectional population survey, who recall their doctors informing them of their familial DM risk were more likely to state that they have made lifestyle changes, participated in DM screening, and have awareness of risk than non-informed individuals. Also, doctors were more likely to inform patients with stronger DM family histories. Identifying this higher risk group, either in isolation or in combination with other recognised risk factors, offers doctors the opportunity to target limited health promotion resources efficiently for primary DM prevention.

## List of Abbreviations

CI: Confidence Interval; DM: Diabetes Mellitus; OR: Odds Ratio.

## Competing interests

The author(s) declare that they have no competing interests.

## Authors' contributions

NQ analysed the dataset and wrote the original manuscript. JK scrutinized the results and edited the manuscript for publication.

## Pre-publication history

The pre-publication history for this paper can be accessed here:



## Supplementary Material

Additional File 1 Diabetes family history section of the *Health Styles 2004 *survey. Section of the *Health Styles 2004 *survey with the family history of Diabetes questions Click here for file
