# Supplementary material for: Informing patients of familial diabetes mellitus risk: How do they respond? A cross-sectional survey
Source: BMC Health Serv Res. 2008 Feb 7;8:37. doi: 10.1186/1472-6963-8-37 (PMC2275238; doi:10.1186/1472-6963-8-37)
Supplement: Additional File 1 — Diabetes family history section of the Health Styles 2004 survey. Section of the Health Styles 2004 survey with the family history of Diabetes questions [file 1472-6963-8-37-S1.doc]

**Additional file 1**

**Diabetes family history section of the *Health Styles 2004* survey**

| **(1)** Has your doctor ever told you that you have a greater chance of getting diabetes because it runs in your family ? |
| --- |
| **(2)** Have you made any lifestyle changes, like diet or exercise, to reduce your chances of getting diabetes ? |
| **(3)** Have you ever been diagnosed by your doctor as having type 2 diabetes? |
| **(4)** Has your mother ever been diagnosed as having type 2 diabetes ? |
| **(5)** Has your father ever been diagnosed as having type 2 diabetes ? |
| **(6)** Have you ever actively collected health information from your relatives for purposes of developing a family health history ? |

| **(7)** How many of your brothers and sisters have been diagnosed with type 2 diabetes? |
| --- |
| **(8)** How many of your mother’s relatives (her sisters, brothers, and parents) were diagnosed with type 2 diabetes ? |
| **(9)** How many of your father’s relatives (his sisters, brothers and parents) were diagnosed with type 2 diabetes ? |
